# Consumption of ultra-processed foods in Brazil: distribution and temporal evolution 2008–2018

**DOI:** 10.11606/s1518-8787.2023057004744

**Published:** 2023-03-15

**Authors:** Maria Laura da Costa Louzada, Gabriela Lopes da Cruz, Karina Augusta Aparecida Nogueira Silva, Ana Giulia Forjaz Grassi, Giovanna Calixto Andrade, Fernanda Rauber, Renata Bertazzi Levy, Carlos Augusto Monteiro

**Affiliations:** I Universidade de São Paulo Faculdade de Saúde Pública Departamento de Nutrição São Paulo SP Brasil Universidade de São Paulo. Faculdade de Saúde Pública. Departamento de Nutrição. São Paulo, SP, Brasil; II Universidade de São Paulo Núcleo de Pesquisas Epidemiológicas em Nutrição e Saúde São Paulo SP Brasil Universidade de São Paulo. Núcleo de Pesquisas Epidemiológicas em Nutrição e Saúde. São Paulo, SP, Brasil; III Universidade de São Paulo Faculdade de Medicina Departamento de Medicina Preventiva São Paulo SP Brasil Universidade de São Paulo. Faculdade de Medicina. Departamento de Medicina Preventiva. São Paulo, SP, Brasil

**Keywords:** Eating, Ultra-Processed Foods, Socioeconomic Factors, Diet, Food, and Nutrition

## Abstract

**OBJECTIVE:**

To evaluate sociodemographic factors associated with the consumption of ultra-processed foods and the temporal evolution of their consumption in Brazil between 2008 and 2018.

**METHODS:**

The study used food consumption data of individuals aged ≥ 10 years from 2008–2009 and 2017–2018 *Pesquisas de Orçamentos Familiares* (POF - Household Budget Surveys), grouping the foods according to the Nova classification. We used crude and adjusted linear regression models to assess the association between sociodemographic characteristics and consumption of ultra-processed foods in 2017–2018 and the temporal variation in their consumption between 2008 and 2018.

**RESULTS:**

Ultra-processed foods accounted for 19.7% of calories in 2017–2018. The adjusted analysis showed that their consumption was higher in women (versus men) and the South and Southeast regions (versus North) and lower in blacks (versus whites) and rural areas (versus urban), in addition to decreasing with the increased age and increasing with higher education and income. Consumption of ultra-processed foods increased by 1.02 percentage points (pp) from 2008–2009 to 2017–2018. This increase was significantly higher among men (+1.59 pp), black people (+2.04 pp), indigenous (+5.96 pp), in the rural area (+2.43 pp), those with up to 4 years of schooling (+1.18 pp), in the lowest income quintile (+3.54 pp), and the North (+2.95 pp) and Northeast (+3.11 pp) regions. On the other hand, individuals in the highest level of schooling (-3.30 pp) and the highest income quintile (-1.65 pp) reduced their consumption.

**CONCLUSIONS:**

The socioeconomic and demographic segments with the lowest relative consumption of ultra-processed foods in 2017–2018 are precisely those that showed the most significant increase in the temporal analysis, pointing to a trend towards national standardization at a higher level of consumption.

## INTRODUCTION

Malnourishment in all its forms, including undernutrition and obesity, is a leading cause of health issues worldwide. Recently, the health effects of climate change have begun to exacerbate this challenge considerably. Together, these three epidemics – obesity, undernutrition, and climate change – represent what is now called the Global Syndemic, as they have mutually reinforcing effects and have several common determinants, including the underlying changes in the food system that have driven the increase in the consumption of ultra-processed foods^1^.

Ultra-processed foods are typically ready-to-eat industrial formulations made with numerous ingredients often sourced from high-yielding crops, such as sugars and syrups, refined starches, fats, protein isolates, and the remains of intensively farmed animals. They usually contain little or no whole food in their composition, in addition to being high in sugar and fat and lacking in fiber and micronutrients. These formulations are visually attractive, having a seductive aroma and very intense or “irresistible” flavors, using sophisticated combinations of flavorings, dyes, emulsifiers, sweeteners, thickeners, and other additives that modify the sensory attributes. Examples include soft drinks, sweet and salty crackers, instant noodles, ready-to-heat foods, candies, toffees, chocolates, and sausages^2^.

Evidence from systematic reviews and meta-analyses of cross-sectional and longitudinal studies, and a randomized clinical trial has shown the association between the consumption of ultra-processed foods and the risk of obesity, several chronic non-communicable diseases, and all-cause mortality^3^. In addition, recent publications also show that these foods are linked to unprecedented environmental damage, contributing to a large part of greenhouse gas emissions and causing deforestation, soil degradation, and loss of biodiversity^7^. Supported by this evidence, the Dietary Guidelines for the Brazilian Population ( *Guia Alimentar para a População Brasileira* ), published in 2014 by the Ministry of Health, have as a golden rule that “prefer *in natura* or minimally processed foods and their culinary preparations to ultra-processed foods”^8^.

National surveys indicate systematic increases in the share of all categories of ultra-processed foods in the set of food purchases for consumption at home carried out by Brazilian families. Between 2002–2003 and 2017–2018, they increased from 12.6% to 18.4% of the total energy purchased in the country’s households, while fresh or minimally processed foods and cooking ingredients fell by 3.8 and 3.5 percentage points (pp), respectively^9^.

The recent availability of two representative surveys of the Brazilian population, with simultaneous information on individual food consumption and sociodemographic characteristics, allows for an unprecedented estimate of the distribution and trend of real consumption of ultra-processed foods in Brazil. Therefore, the objective of this study was to evaluate sociodemographic factors associated with the consumption of ultra-processed foods and the temporal evolution of their consumption in Brazil between 2008 and 2018.

## METHODS

### Data Sources

The data analyzed comprise the individual food consumption module of the *Pesquisas de Orçamentos Familiares* (POF - Household Budget Surveys) carried out by the Brazilian Institute of Geography and Statistics (IBGE) between May 2008 and May 2009 and between July 2017 and July 2018^9 , 10^. The surveys used a complex sampling plan by conglomerates in two stages, involving a random selection of census tracts in the first stage and households in the second. The census tracts come from the IBGE master sample, grouped into strata of households with high geographic and socioeconomic homogeneity. Strata construction considers the geographic location of the sector, household status (urban/rural), and spectrum of socioeconomic variation, defined by the income of the individual responsible for the household. The survey, distributed over the four quarters of the year, resulted in estimates representative of the country as a whole, whether according to the five major regions (North, Northeast, Midwest, Southeast, and South), the household area, the 27 Federation Units (UF), the nine metropolitan areas, or the 27 state capitals.

In 2008-2009, the food consumption module involved 13,569 households (24.3% of the total 55,970 households) and 34,003 individuals aged ≥ 10 years. In 2017–2018, the same module comprised 46,164 residents aged ≥ 10 years from a subsample of 34.7% of 57,920 households. The subsample of households was randomly selected, inviting all individuals aged ten years or older in the selected households to participate in the individual consumption module. The subsample is also nationally representative and allows the construction of results for the domains of Brazil and Major Regions (North, Northeast, Southeast, South, and Midwest).

### Individual Food Consumption

In 2008–2009, the survey assessed the dietary intake using 24-hour dietary records on two non-consecutive days. The individuals should record all the foods consumed on the day, indicating the time, the amounts consumed in household measures, and the preparation methods. The records also contained a question about the custom of adding sugar or artificial sweeteners to beverages, with four alternatives: only sugar, only non-caloric artificial sweeteners, both, or neither. In the end, the agent, trained to collect data on food consumption, transcribed the information recorded into the survey’s electronic data entry system.

In 2017–2018, the survey assessed food consumption using two food records on two non-consecutive days applied based on the Automated Multi-Pass Method^11^. Research agents, trained to collect data on food consumption, gathered, in an interview consisting of several stages, information on all foods consumed the day before, amounts in household measures, and type and methods of preparation. For some pre-selected foods (such as coffee, tea, juices, and bread), they requested information on the addition of ingredients such as sugar, sweetener, and olive oil.

Both surveys imputed amounts considered unlikely or not reported based on a matrix of similarities formed by variables considered correlated with the variable amount consumed^9 , 10^.

The surveys harmonized their food codes to allow the comparability of their estimates. The surveys also broke down all culinary preparations, i.e., consumption items that had more than one food in their preparation (for example, rice cooked with oil, salt, and onion), using the standardized recipes of the Brazilian Food Composition Table of the Universidade de São Paulo, Food Research Center, Version 7.0, (Available at http://www.fcf.usp.br/tbca). Next, the consumed amount of each food was transformed into calories, using the information in the same table.

The surveys categorized foods according to the Nova classification into: *in natura* or minimally processed foods, processed culinary ingredients, processed foods, and ultra-processed foods, and into their respective subgroups^2^. The Nova classification considers the characteristics of food processing. The first group comprises *in natura* or minimally processed foods, edible parts of plants or animals, mushrooms, and algae, soon after their separation from nature or when subjected to the removal of inedible parts, dehydration, grinding, pasteurization, freezing, and other processes that do not involve the addition of other substances. The second group comprises processed culinary ingredients, encompassing substances extracted directly from foods in the first group or from nature, such as sugar, salt, oils, and fats. The third group includes processed foods, including industrial items resulting from adding an ingredient from the second group to food from the first, such as jams, preserves, and cheeses. The fourth group includes ultra-processed foods, industrial formulations typically made with many ingredients, often rich in elements from the second group, containing little or no presence of foods from the first group, and characterized by the significant presence of dyes, stabilizers, texturizers, and other additives.

### Socioeconomic and Demographic Variables

Socioeconomic and demographic characteristics were collected using standardized questionnaires. The variables used were: sex (male/female), age (adolescent – 10 to 19 years old; adult – 20 to 59 years old; and elderly – 60 years old or more), race/color (white, black – black and brown grouped –, yellow, and indigenous), schooling (less than four years, from four to eight incomplete years, from eight to twelve incomplete years, and twelve years or older), *per capita* family income (in quintiles), household area (urban/rural area), and country region (North; Northeast; Southeast; South, and Midwest). The total household income divided by the number of residents characterized the per capita income.

### Data Analysis

First, we described the eating pattern of the population in 2017–2018 by distributing the total calories consumed according to Nova’s four major groups and, within these groups, according to selected subgroups. We presented the mean energy consumption and the share percentage in the total energy consumed for these groups and subgroups.

Next, we presented the spatial distribution of the share of ultra-processed foods in the total energy consumed among the 27 UF in 2017–2018 in a heat map. We assessed the association between socioeconomic characteristics and the share of ultra-processed foods in total dietary energy in 2017–2018 using crude and adjusted linear regression analyses, adjusting each sociodemographic variable with the others. We performed linear trend tests to assess the effect of age, schooling, and income as single continuous variables.

Finally, we evaluated the statistical significance of the temporal variation in the share of ultra-processed foods in total energy between 2008–2009 and 2017–2018 using linear regression models for Brazil and according to socioeconomic characteristics. We used multiplicative interaction terms to explore the potential modification of the effect of time on the consumption of ultra-processed foods by socioeconomic features. The estimates considered the complexity of its sample design. We used Stata (StataCorp 15.0) for data analyses.

## RESULTS

The average daily energy consumption in the Brazilian population aged ten years and over in 2017–2018 was 1,754.61 kcal, with more than half (53.25%) coming from *in natura* or minimally processed foods, 15.78% from processed culinary ingredients, 11.28% from processed foods; and 19.69% from ultra-processed foods ( Table 1 ).

**Table 1:** Share of food in the total energy consumed by the Brazilian population aged ten years and over, according to the Nova classification. *Pesquisa de Orçamentos Familiares* , 2017–2018.

Nova classification of food groups and subgroups	Energy kcal		Percentage of energy share %	
	Mean	95%CI	Mean	95%CI
*In natura* or minimally processed foods	914.09	907.45–920.73	53.25	52.97–53.54
Rice	180.90	178.23–183.58	10.54	10.39–10.68
Beef	125.00	123.37–126.63	7.46	7.35–7.57
Beans	108.06	106–110.11	6.22	6.11–6.33
Poultry	94.98	93.57–96.4	5.71	5.63–5.8
Fruits	48.33	47.25–49.42	2.90	2.83–2.96
Milk	47.70	46.6–48.79	2.78	2.72–2.84
Pasta	45.74	44.42–47.06	2.67	2.59–2.74
Pork	43.34	41.7–44.99	2.34	2.26–2.42
Roots and tubers	35.54	34.59–36.48	2.08	2.03–2.13
Vegetables	28.81	28.45–29.17	1.72	1.7–1.74
100% fruit juice	26.28	25.34–27.21	1.48	1.42–1.53
Cassava flour	25.44	24.12–26.76	1.38	1.31–1.44
Eggs	23.24	22.76–23.73	1.38	1.35–1.41
Fish	19.97	18.97–20.97	1.16	1.1–1.22
Corn and other cereals	17.54	16.66–18.42	1.02	0.97–1.07
Coffee and tea	10.71	10.49–10.93	0.64	0.63–0.65
Wheat flour	10.10	9.75–10.44	0.56	0.55–0.58
Other flours^a^	6.33	5.84–6.82	0.36	0.34–0.39
Viscera	4.90	4.35–5.44	0.28	0.25–0.31
Other vegetables	3.95	3.26–4.64	0.20	0.17–0.23
Nuts and seeds	3.20	2.83–3.56	0.17	0.15–0.18
Other^b^	4.05	3.44–4.65	0.21	0.18–0.24
Processed culinary ingredients	277.07	273.74–280.41	15.78	15.64–15.92
Vegetable oil	133.40	131.51–135.3	7.66	7.56–7.75
Sugar	104.98	103–106.97	5.91	5.81–6.01
Butter	17.00	16.16–17.83	0.97	0.92–1.02
Animal fat	7.52	7.08–7.95	0.40	0.38–0.42
Other^c^	14.17	13.27–15.07	0.84	0.8–0.89
Processed foods	204.36	200.77–207.94	11.28	11.1–11.46
Bread	138.23	135.6–140.86	7.86	7.72–8
Cheese	28.87	27.76–29.98	1.60	1.54–1.66
Beer and wine	19.61	17.88–21.34	0.88	0.8–0.97
Salted meats	8.64	7.91–9.36	0.46	0.43–0.5
Fruits preserved in syrup	5.45	4.89–6.01	0.27	0.24–0.29
Other^d^	3.55	3.13–3.98	0.20	0.18–0.23
Ultra-processed foods	359.09	352.98–365.2	19.69	19.39–20
Margarine	47.48	46.32–48.63	2.68	2.62–2.74
Crackers and salty snacks	42.00	40.54–43.47	2.41	2.33–2.49
Bread	33.05	31.83–34.27	1.94	1.86–2.01
Cookies	31.69	30.27–33.12	1.66	1.59–1.74
Cold cuts and sausages	29.99	29.15–30.83	1.71	1.66–1.75
Chocolate, ice cream, and industrialized desserts	29.16	27.66–30.66	1.48	1.41–1.55
Soft drinks	25.80	24.81–26.78	1.40	1.34–1.45
Hot dog, hamburgers and sandwiches	22.03	20.56–23.5	1.18	1.1–1.26
Pizza	20.54	17.2–23.89	1.07	0.83–1.3
Dairy beverages	18.06	17.08–19.05	0.99	0.94–1.05
Ready or semi-ready meals^e^	11.63	10.63–12.63	0.65	0.59–0.7
Fried or baked snacks	11.55	10.44–12.66	0.62	0.56–0.68
Industrialized Juices and other artificial drinks	10.45	9.8–11.1	0.57	0.53–0.6
Ready-made sauces	8.05	7.6–8.5	0.44	0.42–0.47
Sweet cakes and pies	7.37	6.7–8.03	0.40	0.36–0.43
Other^f^	10.24	9.33–11.14	0.50	0.45–0.54
**Total**	**1,754.61**	**1,743.5–1,765.8**	**100.00**	

^a^ Corn flour, oats, and others.

^b^ Includes mushrooms, seafood, and other meats.

^c^ Includes coconut milk, starches, vinegar, and salt.

^d^ Includes canned vegetables/legumes, canned fish, sweetened/salted nuts, nuts and seeds, and tomato paste.

^e^ Includes frozen/ready-to-heat pasta dishes, noodles, soups, and other convenience foods.

^f^ Includes breakfast cereal, cheese, hard liquor, and protein/calorie supplements.

Among *in natura* or minimally processed foods, rice stands out, corresponding to 10.54% of total energy, followed by beef, with 7.46%; beans, with 6.22%; and poultry, with 5.71%. Next, in decreasing order of contribution to the total energy consumed, are fruits (2.90%), milk (2.69%), pasta (2.67%), pork (2.34%), roots and tubers (2.08%), and greens and vegetables (1.72%) ( Table 1 ).

Among the processed culinary ingredients, the group with the most significant contribution to the total amount of energy was vegetable oil (7.66%), followed by sugar (5.91%). Among processed foods, the group with the highest contribution to total energy was bread (7.86%), followed by cheese (1.60%) ( Table 1 ).

Among the ultra-processed foods, margarine stands out, representing 2.68% of the total energy, followed by crackers and salty snacks, with 2.41%; bread, with 1.94%; cookies, with 1.66%; and cold cuts and sausages, with 1.71%. Next, in decreasing order of contribution to the total energy consumed, chocolate, ice cream, and industrialized desserts (1.48%), soft drinks (1.40%), and hot dogs, hamburgers, and sandwiches (1.18%) ( Table 1 ).

The Figure shows the share of ultra-processed foods in the total energy consumed in each UF. In general, the UFs in the South and Southeast regions had the highest average participation of ultra-processed foods in total consumed energy. In contrast, the states in the North and Northeast had the lowest. Rio Grande do Sul (24.06% of the total consumed energy), followed by Santa Catarina (23.23%), and São Paulo (22.38%) presented the most significant shares. The smallest shares were in Piauí (12.58%), Maranhão (13.87%), and Tocantins (13.92%).

**Figure:**
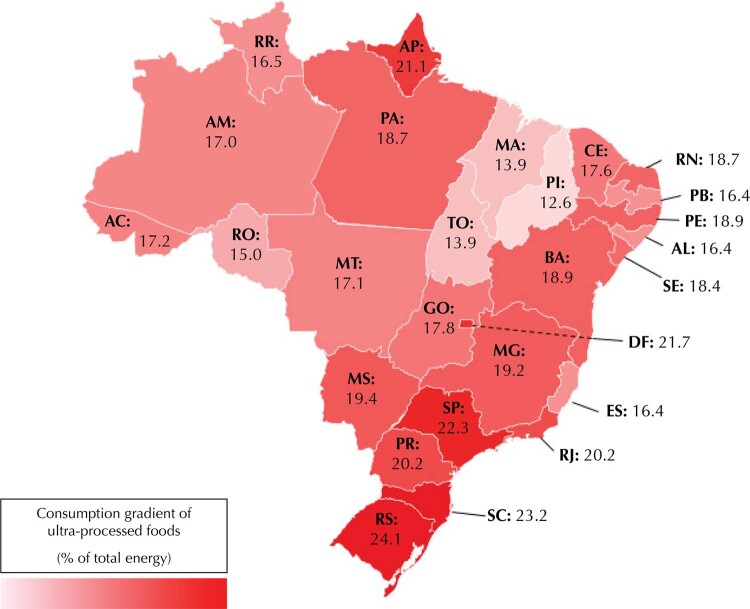
Percentage of share of ultra-processed foods in total calories consumed by the Brazilian population aged ten years and over in each Federative Unit. *Pesquisa de Orçamentos Familiares* , 2017–2018. Federative Units: AM: Amazonas; AP: Amapá; BA: Bahia; CE: Ceará; DF: Distrito Federal; ES: Espírito Santo; GO: Goiás; MA: Maranhão; MG: Minas Gerais; MS: Mato Grosso do Sul; MT: Mato Grosso; PA: Pará; PB: Paraíba; PE: Pernambuco; PI: Piauí; PR: Paraná; RJ: Rio de Janeiro; RN: Rio Grande do Norte; RO: Rondônia; RR: Roraima; RS: Rio Grande do Sul; SC: Santa Catarina; SE: Sergipe; SP: São Paulo; TO: Tocantins.

Table 2 presents the analysis of the association between socioeconomic variables and the share of ultra-processed foods in the total energy consumed by the Brazilian population aged ten years or older. All variables included in the analysis were significantly associated with the share of ultra-processed foods in total energy consumed. In both the crude and adjusted analysis, the caloric share of ultra-processed foods y was significantly higher among women (compared with men), lower among black people (compared with whites), and in rural areas (compared with urban areas), in addition to decreasing with increased age and increasing with higher schooling and income. In the crude analysis, the share of ultra-processed foods was higher in the Southeast, South, and Midwest regions than in the North. However, in the adjusted analysis, the Midwest region had no statistical significance.

**Table 2:** Share of ultra-processed foods in the total calories consumed by the Brazilian population aged ten years and over, according to socioeconomic and demographic variables. *Pesquisa de Orçamentos Familiares* , 2017–2018.

Variables	% energy share of ultra-processed foods					
	Crude mean	Crude coefficient	95%CI	Adjusted mean	Adjusted coefficient^a^	95%CI
Sex						
Male	19.13	Ref.	-	19.10	Ref.	-
Female	20.21	1.08	0.73 to 1.43^c^	20.24	1.15	0.82 to 1.48^c^
Age^b^						
Adolescent	26.49	Ref.	-	29.07	Ref.	-
Adult	19.46	-7.03	-7.61 to -6.44	18.85	-10.22	-10.82 to -9.61
Elderly	15.11	-11.38	-12.1 to -10.65^d^	15.41	-13.66	-14.42 to -12.91^d^
Race/color						
White	21.26	Ref.	-	20.15	Ref.	-
Black (Black and Brown)	18.44	-2.82	-3.36 to -2.28^c^	19.31	-0.84	-1.38 to -0.30^c^
Yellow	22.46	1.20	-1.54 to 3.94	20.73	0.58	-1.64 to 2.79
Indigenous	20.75	-0.51	-3.77 to 2.75	21.51	1.35	-1.46 to 4.16
Years of schooling						
Less than 4 years	13.40	Ref.	-	16.43	Ref.	-
From 4 to 8 incomplete years	18.25	4.84	4.3 to 5.3	17.68	1.26	0.76 to 1.75
From 8 to 12 incomplete years	20.24	6.83	6.2 to 7.4	19.14	2.72	2.12 to 3.31
12 years or older	22.17	8.77	8.1 to 9.4^d^	22.18	5.75	5.13 to 6.38^d^
*Per capita* family income quintiles						
1	16.85	Ref.	-	17.30	Ref.	-
2	18.17	1.32	0.66 to 1.98	18.45	1.14	0.49 to 1.79
3	19.63	2.78	1.73 to 3.84	19.99	2.68	1.71 to 3.66
4	20.71	3.86	3.06 to 4.67	20.64	3.33	2.52 to 4.15
5	23.10	6.25	5.46 to 7.04^d^	22.10	4.80	3.92 to 5.67^d^
Household status						
Urban	20.55	Ref.	-	20.16	Ref.	-
Rural	14.65	-5.90	-6.43 to -5.36^c^	16.96	-3.20	-3.66 to -2.74^c^
Country region						
North	17.52	Ref.	-	18.42	Ref.	-
Northeast	17.35	-0.17	-0.9 to 0.56	18.84	0.42	-0.31 to 1.15
Southeast	20.86	3.34	2.44 to 4.23^c^	20.11	1.69	0.76 to 2.61^c^
South	22.42	4.89	4.01 to 5.78^c^	21.76	3.34	2.41 to 4.27^c^
Midwest	18.65	1.13	0.24 to 2.02^c^	17.86	-0.56	-1.46 to 0.35

^a^ Linear regression analysis with all variables included in the model simultaneously.

^b^ Adolescent (10 to 19 years old), adult (20 to 59 years old), and elderly (over 60 years old).

^c^ p ≤ 0.005 in comparison with the reference category.

^d^ p of linear trend < 0.001.

The contribution of ultra-processed foods to total energy increased significantly in the ten years between the two surveys (from 18.67% to 19.69%) ( Table 3 ).

**Table 3:** Temporal evolution of the share of ultra-processed foods in the total energy consumed (%) by the Brazilian population aged ten years and over according to socioeconomic and demographic strata. *Pesquisas de Orçamentos Familiares* 2008–2009 and 2017–2018.

	Share in total calories consumed (%)		Coefficient	95%CI
	Mean			
	2008–2009	2017–2018		
Brazil	18.67	19.69	1.02	0.69 to 1.35^b^
Sex				
Male	17.54	19.13	1.59	1.1 to 2.08^b^
Female	19.73	20.21	0.49	0.04 to 0.94^b^
Ageª				
Adolescent	25.10	26.49	1.39	0.55 to 2.23^b^
Adult	18.12	19.46	1.34	0.94 to 1.74^b^
Elderly	13.14	15.11	1.97	1.25 to 2.69^b^
Race/color				
White	21.07	21.26	0.19	-0.32 to 0.69
Black (Black and Brown)	16.39	18.44	2.04	1.61 to 2.48^b^
Yellow	17.89	22.46	4.57	-0.23 to 9.37
Indigenous	14.79	20.75	5.96	1.54 to 10.39^b^
Years of schooling				
Less than 4 years	12.23	13.40	1.18	0.63 to 1.73^b^
From 4 to 8 incomplete years	17.64	18.25	0.61	0.03 to 1.18^b^
From 8 to 12 incomplete years	20.91	20.24	-0.67	-1.3 to -0.04^b^
12 years or older	25.47	22.17	-3.30	-4.18 to -2.42^b^
*Per capita* family income quintiles				
1	13.31	16.85	3.54	3.01 to 4.07^b^
2	16.56	18.17	1.61	0.93 to 2.28^b^
3	18.08	19.63	1.56	0.77 to 2.35^b^
4	20.66	20.71	0.06	-0.71 to 0.83
5	24.75	23.10	-1.65	-2.47 to -0.83^b^
Household status				
Urban	19.94	20.55	0.60	0.23 to 0.98^b^
Rural	12.22	14.65	2.43	2 to 2.87^b^
Regions				
North	14.57	17.52	2.95	2.31 to 3.59^b^
Northeast	14.24	17.35	3.11	2.71 to 3.51^b^
Southeast	21.03	20.86	-0.17	-0.81 to 0.47
South	22.64	22.42	-0.22	-0.94 to 0.5
Midwest	17.77	18.65	0.87	0.01 to 1.75^b^

^a^ Adolescent (10 to 19 years old), adult (20 to 59 years old), and elderly (over 60 years old).

^b^ p of heterogeneity < 0.05.

A statistically significant interaction occurred between the year of study and the variables sex, race/color, schooling, income, household status, and region. The increase in the share of ultra-processed foods in the total consumed energy was statistically significant in both sexes but more expressive in men (from 17.54% to 19.13%) than in women (from 19.73% to 20.21%). In contrast, the share of ultra-processed foods in the total energy consumed increased significantly among black people (from 16.39% to 18.44%) and indigenous people (from 14.79% to 20.75%), but not between white and yellow people ( Table 3 ).

Consumption of ultra-processed foods increased significantly among people with up to four and between four and eight years of schooling (from 12.23% to 13.40% and from 17.64% to 18.25%, respectively). On the other hand, a slight reduction in its consumption occurred among those with eight to twelve years of schooling (from 20.91% to 20.24%) and a more significant decrease in the highest level of education (from 25.47% to 22.17%). Similarly, the consumption of ultra-processed foods increased significantly in the three lowest quintiles of family income (from 13.31% to 16.85% in the 1^st^, from 16.56% to 18.17% in the 2^nd^, and from 18.08% to 19.63% in the 3^rd^) and significantly reduced in the highest quintile (from 24.75% to 23.10%), not changing in the 4^th^ ( Table 3 ).

The increase in the share of ultra-processed foods in total consumed energy was statistically significant in rural and urban areas but more intense in the former (from 12.22% to 14.65%) than in the latter (from 19.94% to 20 .55%). Finally, consumption of ultra-processed foods increased significantly in the North (from 14.57% to 17.52%) and Northeast (from 14.24% to 17.35%) regions, rising slightly in the Midwest region (from 17.77% to 18.65%). On the other hand, values remained unchanged in the South and Southeast ( Table 3 ).

## DISCUSSION

This study showed that ultra-processed foods represented around 20% of the total energy consumed in 2017–2018, using recent and representative data from the Brazilian population. However, this consumption varied significantly according to socioeconomic and demographic strata, being higher among women, adolescents, white people, individuals with higher income and schooling, and residents of urban areas and in the South and Southeast regions. In addition, the results showed an average increase of 5.5% in the consumption of ultra-processed foods over ten years. Such growth was more expressive in black and indigenous people, residents of rural areas and in the North and Northeast regions, and population groups with lower levels of schooling and income.

The inverse association between the consumption of ultra-processed foods and age observed in Brazil reflects a global pattern. Countries such as the United Kingdom, the United States, Canada, Chile, Colombia, and Mexico^12^ also show higher consumption of ultra-processed foods among children and adolescents, highlighting the vulnerability of this population group to increasing exposure and easy access to these foods. On the other hand, studies are not so consistent when analyzing socioeconomic status. Population-based studies conducted in Latin American countries showed results similar to those observed in Brazil. In Chile, individuals residing in urban areas, in the metropolitan region, and with higher income had significantly higher consumption of ultra-processed foods^13^. In Mexico, consumption of ultra-processed foods increased with economic and educational level^s15^. In Colombia, individuals from urban areas with high socioeconomic status had 1.5 to 1.7 times higher energy intake of ultra-processed foods compared with those from lower socioeconomic status and residents of rural regions^14^. In contrast, in higher-income countries, such as the United States, United Kingdom, and Canada, only minor differences appeared in the consumption of ultra-processed foods between population strata. This result reflects how these foods, representing more than 50% of the total energy consumed by these populations, permeated and reached all social strata, standardizing eating habits^12 , 16 , 17^.

Despite the slight difference in magnitude, we also observed lower consumption of ultra-processed foods among blacks and browns (compared to whites), even after adjusting for other sociodemographic variables. Food consumption results from the interaction of different factors. Cultural, historical, and psychological characteristics closely linked to ethnic-racial issues and racism influence food consumption. Although our results indicate that blacks and browns adhered more to the golden rule of the Dietary Guidelines for the Brazilian Population, it is essential to point out that this does not necessarily represent a higher overall diet quality. Other studies have already indicated that this difference is due almost entirely to the higher consumption of basic foods such as rice and beans by black and brown people. However, the same does not happen for other *in natura* or minimally processed foods such as fruits, vegetables, whole graind, nuts, and seeds^18^.

Temporal evolution-related analyses show that the socioeconomic and demographic segments with the lowest consumption of ultra-processed foods in 2017–2018 are precisely those that showed the most significant increase in the period evaluated. Therefore, the expansion of their access by socially more vulnerable groups explains the growth in the consumption of ultra-processed foods in the country. It is due to the reduction in the relative prices of these foods, the development of their offer in most diverse shopping places, and the growing penetration of international industries in more remote areas of the country^19^. Analyses of data from the National System of Consumer Price Indexes show that, although ultra-processed foods are still more expensive than *in natura* or minimally processed foods and culinary ingredients, their price has decreased gradually and substantially since the beginning of the 2000s^21^. Similarly, in recent years, there has been an increase in food purchases in supermarket chains, which offer a higher concentration of ultra-processed foods compared with other, more traditional shopping places^22^. Direct and specific advertising of ultra-processed foods to lower-income communities has also helped to accelerate their growth in these segments of society^19 , 20^.

On the other hand, there was a tendency towards stagnation in the consumption of ultra-processed foods in more privileged socioeconomic strata. One hypothesis for this phenomenon is, in the most recent period, the increase in the dissemination of information about the harmful effects of ultra-processed foods (mainly some types, such as sugar-sweetened beverages). Such information is more accessible to people with better socioeconomic conditions^23^. It is also worth remembering that the Dietary Guidelines for the Brazilian Population, which assertively recommend avoiding the consumption of ultra-processed foods, was published in 2014 and quickly reverberated in several media outlets.

The consumption of ultra-processed foods has been growing in many countries. Household food purchase data also showed, for example, that in Canada, the contribution of ultra-processed foods increased from 24.4% in 1938–1939 to 54.9% in 2001, and in Mexico from 10.5% in 1984 to 23.1% in 2016^24 , 25^. More recently, analyses of retail food sales databases in 80 countries showed a significant rise in sales of ultra-processed foods between 2002 and 2016, with particular acceleration among middle-income countries. This increase was positively associated with the temporal evolution of the body mass index of populations^26^.

Therefore, the search for strategies to reduce or slow down the expansion of consumption of ultra-processed foods is mandatory. Possible measures include taxation and intervention in the price of ultra-processed products, regulation of advertisements and commercial promotions, especially those aimed at children and young people, and adequate nutritional labeling. Food and nutrition education actions and policies should adopt those measures to stimulate the production and sale of *in natura* and minimally processed foods, aiming at greater accessibility by all population segments^20^. The apparent drop in the consumption of ultra-processed foods in the higher-income population stratum (data not recorded in any previous edition of the POF) suggests that the social norm concerning the consumption of these foods has been changing. However, future data are essential to confirm this trend.

Among the strengths of this study, the following stand out: the rigorously probabilistic nature of the samples studied and national representativeness, ensured by the study of more than 30 thousand people residing in urban and rural areas of the various regions of the country, data collection from two days of food consumption through validated software, and provision of a database with more than 1,200 food items. On the other hand, this study has limitations arising from potential biases inherent in the use of dietary surveys: underestimation/overestimation of the consumption of certain food groups, differences between actual culinary recipes and standardized recipes, and differences between the exact nutritional composition of consumed foods and the one indicated by the nutritional composition table used. To minimize part of these biases, IBGE pre-tested and validated the collection instruments, performed quality control procedures during data collection, and deleted inconsistent records, replacing them with imputed values. In addition, the table of the nutritional composition of foods used is specific for the Brazilian population with strict quality control. Finally, another possible limitation relates to the different methods used to collect food consumption information in the two surveys. Despite this, such a change had little effect on the estimation of diet composition, enabling us to compare the two banks with the harmonization strategies used^27^.

In conclusion, this study described the sociodemographic distribution and growth in the consumption of ultra-processed foods in Brazil. The population segments with the lowest relative consumption of these foods in 2017–2018 are precisely those showing a more significant increase in the temporal analysis, pointing to a trend of national standardization at a higher level of consumption and, therefore, with increased risks to population health.
