# Supplementary material for: Consumption of ultra-processed foods in Brazil: distribution and temporal evolution 2008–2018
Source: Rev Saude Publica. 2023 Mar 15;57:12. doi: 10.11606/s1518-8787.2023057004744 (PMC10118420; doi:10.11606/s1518-8787.2023057004744)
Supplement: Translation

# Translation: Consumo de alimentos ultraprocessados no Brasil: distribuição e evolução temporal 2008–2018

**Authors:** Maria Laura da Costa Louzada, Gabriela Lopes da Cruz, Karina Augusta Aparecida Nogueira Silva, Ana Giulia Forjaz Grassi, Giovanna Calixto Andrade, Fernanda Rauber, Renata Bertazzi Levy, Carlos Augusto Monteiro

**Affiliations:** I Brasil Universidade de São Paulo. Faculdade de Saúde Pública. São Paulo, SP, Brasil; II Brasil Universidade de São Paulo. Núcleo de Pesquisas Epidemiológicas em Nutrição e Saúde. São Paulo, SP, Brasil; III Brasil Universidade de São Paulo. Faculdade de Medicina. São Paulo, SP, Brasil

**Keywords:** Ingestão de Alimentos, Alimentos Ultraprocessados, Fatores Socioeconômicos, Alimentos, Dieta e Nutrição

## Abstract

**OBJETIVO:**

Avaliar fatores sociodemográficos associados ao consumo de alimentos ultraprocessados e a evolução temporal do consumo no Brasil entre 2008 e 2018.

**MÉTODOS:**

Foram utilizados dados do consumo alimentar de indivíduos com idade ≥ 10 anos das Pesquisas de Orçamentos Familiares (POF) 2008–2009 e 2017–2018. Os alimentos foram agrupados segundo a classificação Nova. Modelos de regressão linear brutos e ajustados foram utilizados para avaliar a associação entre características sociodemográficas e o consumo de ultraprocessados em 2017–2018 e a variação temporal de seu consumo entre 2008 e 2018.

**RESULTADOS:**

Alimentos ultraprocessados representaram 19,7% das calorias em 2017–2018. A análise ajustada mostrou que seu consumo foi maior no sexo feminino ( *versus* masculino) e nas regiões Sul e Sudeste ( *versus* Norte), e menor em negros ( *versus* brancos) e na área rural ( *versus* urbana), além de diminuir com o aumento da idade e aumentar com escolaridade e renda. O consumo de ultraprocessados aumentou 1,02 pontos percentuais (pp) de 2008–2009 a 2017–2018, sendo este aumento mais expressivo em homens (+1,59 pp), negros (+2,04 pp), indígenas (+5,96 pp), na área rural (+2,43 pp), naqueles com até 4 anos de estudo (+1,18 pp), no quinto mais baixo de renda (+3,54 pp) e nas regiões Norte (+2,95 pp) e Nordeste (+3,11 pp). Por outro lado, seu consumo se reduziu na maior faixa de escolaridade (-3,30 pp) e no quinto mais alto de renda (-1,65 pp).

**CONCLUSÕES:**

Os segmentos socioeconômicos e demográficos que tiveram menor consumo relativo de ultraprocessados em 2017–2018 são justamente os que apresentaram um aumento mais expressivo na análise temporal, apontando para uma tendência de padronização nacional em um patamar de consumo mais alto.

## INTRODUÇÃO

A má-nutrição em todas as suas formas, incluindo desnutrição e obesidade, é a principal causa de problemas de saúde em todo o mundo. Recentemente, os efeitos das mudanças climáticas na saúde começaram a exacerbar consideravelmente esse desafio. Juntas, essas três epidemias – obesidade, desnutrição e mudanças climáticas – representam o que agora é chamado de Sindemia Global, visto que causam efeitos que potencializam umas às outras e possuem vários determinantes comuns, incluindo as mudanças subjacentes do sistema alimentar que impulsionaram o aumento do consumo de alimentos ultraprocessados^1^.

Alimentos ultraprocessados são formulações industriais tipicamente prontas para consumo, feitas com inúmeros ingredientes frequentemente obtidos a partir de colheitas de alto rendimento, como açúcares e xaropes, amidos refinados, gorduras, isolados proteicos, além de restos de animais de criação intensiva. Eles usualmente contêm pouco ou nenhum alimento inteiro em sua composição, além de serem fartos em açúcar e gorduras e carentes de fibras e micronutrientes. Essas formulações são feitas para serem visualmente atrativas, terem aroma sedutor e sabores muito intensos ou “irresistíveis”, usando para tais finalidades combinações sofisticadas de flavorizantes, corantes, emulsificantes, edulcorantes, espessantes e outros aditivos que modificam os atributos sensoriais. Exemplos incluem refrigerantes, biscoitos de pacote doces e salgados, macarrão instantâneo, alimentos prontos para aquecer, doces, balas, chocolates e embutidos^2^.

Evidências provenientes de revisões sistemáticas e meta-análises de estudos transversais e longitudinais, além de um ensaio clínico randomizado, têm evidenciado a associação entre o consumo de alimentos ultraprocessados e o risco de obesidade e de diversas doenças crônicas não transmissíveis, além de mortalidade por todas as causas^3^. Além disso, publicações recentes também mostram que esses alimentos estão relacionados a danos ambientais sem precedentes, contribuindo com grande parte das emissões de gases de efeito estufa e causando desmatamento, degradação do solo e perda de biodiversidade^7^. Corroborado por essas evidências, o Guia Alimentar para a População Brasileira, publicado em 2014 pelo Ministério da Saúde, traz como regra de ouro que se “prefiram alimentos *in natura* ou minimamente processados e suas preparações culinárias a alimentos ultraprocessados”^8^.

Pesquisas nacionais indicam aumentos sistemáticos na participação de todas as categorias de alimentos ultraprocessados no conjunto das compras de alimentos para consumo dentro do domicílio realizadas pelas famílias brasileiras. Entre 2002–2003 e 2017–2018, eles passaram de 12,6% para 18,4% do total de energia adquirida nos domicílios do país, enquanto os alimentos *in natura* ou minimamente processados e os ingredientes culinários caíram respectivamente 3,8 e 3,5 pontos percentuais (pp)^9^.

A recente disponibilidade de dois inquéritos representativos da população brasileira, com informações simultâneas sobre consumo alimentar individual e características sociodemográficas, permite realizar a estimativa inédita da distribuição e da tendência do consumo efetivo de alimentos ultraprocessados no Brasil. Sendo assim, o objetivo deste estudo foi avaliar fatores sociodemográficos associados ao consumo de alimentos ultraprocessados e a evolução temporal do consumo no Brasil entre 2008 e 2018.

## MÉTODOS

### Fontes de Dados

Os dados analisados compõem o módulo de consumo alimentar pessoal das Pesquisas de Orçamentos Familiares (POF) realizadas pelo Instituto Brasileiro de Geografia e Estatística (IBGE) entre maio de 2008 e maio de 2009 e entre julho de 2017 e julho de 2018^9 , 10^. As pesquisas utilizaram um plano amostral complexo por conglomerados em dois estágios, envolvendo sorteio dos setores censitários no primeiro estágio e domicílios no segundo. Os setores censitários são provenientes de amostra mestre do IBGE, agrupados em estratos de domicílios com alta homogeneidade geográfica e socioeconômica. A construção dos estratos considerou a localização geográfica do setor, a situação do domicílio (urbana/rural) e o espectro de variação socioeconômica, definido através da renda do indivíduo responsável pelo domicílio. A pesquisa foi distribuída ao longo dos quatro trimestres do ano. As estimativas obtidas nas pesquisas são representativas para o país como um todo, seja de acordo com as cinco grandes regiões (Norte, Nordeste, Centro-Oeste, Sudeste e Sul), a situação do domicílio, as 27 Unidades da Federação (UF), as nove regiões metropolitanas ou as 27 capitais dos estados.

Em 2008-2009, o módulo de consumo alimentar envolveu 13.569 domicílios (24,3% do total de 55.970 domicílios) e 34.003 indivíduos com ≥ 10 anos de idade. Em 2017–2018, o mesmo módulo compreendeu 46.164 moradores com ≥ 10 anos de idade de uma subamostra de 34,7% dos 57.920 domicílios. A subamostra de domicílios foi selecionada de forma aleatória e todos os indivíduos com 10 anos ou mais de idade nos domicílios selecionados foram convidados a participar do módulo de consumo individual. A subamostra também possui representatividade nacional e permite a construção de resultados para os domínios Brasil e Grandes Regiões (Norte, Nordeste, Sudeste, Sul e Centro-Oeste).

### Consumo Alimentar Individual

Em 2008–2009, o consumo alimentar foi avaliado por meio de registros alimentares de 24 horas em dois dias não consecutivos. Os indivíduos foram solicitados a registrar todos os alimentos consumidos no dia, indicando o horário, as quantidades consumidas em medidas caseiras e a forma de preparo. Os registros continham ainda uma pergunta sobre o costume de adicionar açúcar ou adoçantes artificiais às bebidas, admitindo-se quatro alternativas para a resposta: apenas açúcar, apenas adoçantes artificiais não calóricos, ambos ou nenhum dos dois. Ao final, o agente, treinado para coleta de dados de consumo alimentar, realizava a transcrição das informações registradas para o sistema eletrônico de entrada de dados da pesquisa.

Em 2017–2018, o consumo alimentar foi avaliado por meio de dois registros alimentares em dois dias não consecutivos, aplicados com base no Método Automatizado de Múltiplos Passos^11^. Agentes de pesquisa, treinados para coleta de dados de consumo alimentar, coletaram, em entrevista composta de diversos estágios, informações sobre todos os alimentos consumidos no dia anterior, quantidades em medidas caseiras, tipo e métodos de preparação. Para alguns alimentos pré-selecionados (como café, chá, sucos e pães), solicitaram-se informações sobre adição de ingredientes como açúcar, adoçante e azeite.

Nas duas pesquisas, quantidades consideradas improváveis ou não informadas foram imputadas com base em matriz de similaridades formadas por variáveis consideradas correlacionadas com a variável quantidade consumida^9 , 10^.

Os códigos dos alimentos presentes nas duas pesquisas foram harmonizados com o objetivo de permitir a comparabilidade das suas estimativas. Todas as preparações culinárias, ou seja, itens de consumo que possuíam mais de um alimento na sua preparação (por exemplo, arroz cozido com óleo, sal e cebola), foram desagregadas utilizando-se as receitas padronizadas da Tabela Brasileira de Composição de Alimentos da Universidade de São Paulo, Food Research Center, Versão 7.0, (Disponível em: http://www.fcf.usp.br/tbca). A seguir, a quantidade consumida de cada alimento foi transformada em calorias, empregando-se as informações da mesma tabela.

Os alimentos foram categorizados de acordo com a classificação Nova em: alimentos *in natura* ou minimamente processados, ingredientes culinários, alimentos processados e alimentos ultraprocessados, e em respectivos subgrupos^2^. A classificação Nova leva em consideração as características do processamento alimentar. O primeiro grupo compreende alimentos *in natura* ou minimamente processados, sendo eles partes comestíveis de plantas ou animais, cogumelos e algas, logo após sua separação da natureza ou quando submetidos à remoção de partes não comestíveis, desidratação, moagem, pasteurização, congelamento e demais processos que não envolvam a adição de outras substâncias. O segundo grupo é composto por ingredientes culinários, englobando substâncias extraídas diretamente de alimentos do primeiro grupo ou da natureza, tais como açúcar, sal, óleos e gorduras. O terceiro grupo é formado por alimentos processados, incluindo itens industriais resultantes da adição de um ingrediente do segundo grupo a um alimento do primeiro, tais como compotas, conservas e queijos. O quarto grupo é o de alimentos ultraprocessados, formulações industriais feitas tipicamente com muitos ingredientes, frequentemente ricos em ingredientes do segundo grupo, contendo pouca ou nenhuma presença de alimentos do primeiro grupo e caracterizados pela presença marcante de corantes, estabilizantes, texturizantes e demais aditivos.

### Variáveis Socioeconômicas e Demográficas

As características socioeconômicas e demográficas foram coletadas por meio de questionários padronizados. As variáveis utilizadas foram: sexo (masculino/feminino), idade (adolescente – 10 a 19 anos; adulto – 20 a 59 anos; e idoso – 60 anos ou mais), raça/cor (branca, negra – preta e parda agrupadas –, amarela e indígena), escolaridade (menos de quatro anos, de quatro a oito anos incompletos, de oito a doze anos incompletos e doze anos ou mais), renda familiar *per capita* (em quintos), situação do domicílio (área urbana/rural) e região do país (Norte; Nordeste; Sudeste; Sul e Centro-Oeste). A renda *per capita* foi calculada como a renda total do domicílio dividida pelo número de moradores.

### Análise de Dados

Primeiramente, o padrão da alimentação da população em 2017–2018 foi descrito distribuindo o total de calorias consumidas segundo os quatro grandes grupos da Nova e, internamente a esses grupos, segundo subgrupos selecionados. Foram apresentadas médias de consumo de energia e o percentual de participação no total de energia consumida para cada um desses grupos e subgrupos.

A seguir, a distribuição espacial da participação de alimentos ultraprocessados no total de energia consumida entre as 27 UF em 2017–2018 foi apresentada em um mapa de calor. A associação entre as características socioeconômicas e a participação de alimentos ultraprocessados no total de energia da dieta em 2017–2018 foi avaliada por meio de análises de regressão linear brutas e ajustadas, em que cada variável sociodemográfica foi ajustada pelas demais. Testes de tendência linear foram realizados a fim de avaliar o efeito da idade, escolaridade e renda como variáveis contínuas únicas.

Por fim, o significado estatístico da variação temporal da participação de alimentos ultraprocessados no total de energia entre 2008–2009 e 2017–2018 foi avaliado por meio de modelos de regressão linear para o Brasil como um todo e segundo características socioeconômicas. Termos de interação multiplicativos foram usados para explorar a potencial modificação do efeito do tempo no consumo de alimentos ultraprocessados pelas características socioeconômicas. As estimativas levaram em conta a complexidade de seu desenho amostral. As análises foram feitas no Stata (StataCorp 15.0).

## RESULTADOS

O consumo médio diário de energia na população brasileira com idade igual ou superior a 10 anos de idade em 2017–2018 foi de 1.754,61 kcal, sendo mais da metade (53,25%) proveniente de alimentos *in natura* ou minimamente processados; 15,78%, de ingredientes culinários processados; 11,28%, de alimentos processados; e 19,69%, de alimentos ultraprocessados ( Tabela 1 ).

**Tabela 1:** Participação dos alimentos no total de energia consumida pela população brasileira com 10 anos ou mais de idade, segundo a classificação Nova. Pesquisa de Orçamentos Familiares, 2017–2018.

Grupos e subgrupos de alimentos da classificação Nova	Energia kcal		Percentual de participação calórica %	
	Média	IC95%	Média	IC95%
**Alimentos in natura ou minimamente processados**	**914,09**	**907,45–920,73**	**53,25**	**52,97–53,54**
Arroz	180,90	178,23–183,58	10,54	10,39–10,68
Carne bovina	125,00	123,37–126,63	7,46	7,35–7,57
Feijão	108,06	106–110,11	6,22	6,11–6,33
Carne de aves	94,98	93,57–96,4	5,71	5,63–5,8
Frutas	48,33	47,25–49,42	2,90	2,83–2,96
Leite	47,70	46,6–48,79	2,78	2,72–2,84
Macarrão	45,74	44,42–47,06	2,67	2,59–2,74
Carne suína	43,34	41,7–44,99	2,34	2,26–2,42
Raízes e tubérculos	35,54	34,59–36,48	2,08	2,03–2,13
Verduras e legumes	28,81	28,45–29,17	1,72	1,7–1,74
Suco de fruta natural	26,28	25,34–27,21	1,48	1,42–1,53
Farinha de mandioca	25,44	24,12–26,76	1,38	1,31–1,44
Ovos	23,24	22,76–23,73	1,38	1,35–1,41
Peixes	19,97	18,97–20,97	1,16	1,1–1,22
Milho e outros cereais	17,54	16,66–18,42	1,02	0,97–1,07
Café e chá	10,71	10,49–10,93	0,64	0,63–0,65
Farinha de trigo	10,10	9,75–10,44	0,56	0,55–0,58
Outras farinhas^a^	6,33	5,84–6,82	0,36	0,34–0,39
Vísceras em geral	4,90	4,35–5,44	0,28	0,25–0,31
Outras leguminosas	3,95	3,26–4,64	0,20	0,17–0,23
Castanhas, nozes e sementes	3,20	2,83–3,56	0,17	0,15–0,18
Outros^b^	4,05	3,44–4,65	0,21	0,18–0,24
**Ingredientes culinários processados**	**277,07**	**273,74–280,41**	**15,78**	**15,64–15,92**
Óleo vegetal	133,40	131,51–135,3	7,66	7,56–7,75
Açúcar	104,98	103–106,97	5,91	5,81–6,01
Manteiga	17,00	16,16–17,83	0,97	0,92–1,02
Banha	7,52	7,08–7,95	0,40	0,38–0,42
Outros^c^	14,17	13,27–15,07	0,84	0,8–0,89
**Alimentos processados**	**204,36**	**200,77–207,94**	**11,28**	**11,1–11,46**
Pães	138,23	135,6–140,86	7,86	7,72–8
Queijos	28,87	27,76–29,98	1,60	1,54–1,66
Cerveja e vinho	19,61	17,88–21,34	0,88	0,8–0,97
Carnes salgadas	8,64	7,91–9,36	0,46	0,43–0,5
Doce de frutas	5,45	4,89–6,01	0,27	0,24–0,29
Outros^d^	3,55	3,13–3,98	0,20	0,18–0,23
**Alimentos ultraprocessados**	**359,09**	**352,98–365,2**	**19,69**	**19,39–20**
Margarina	47,48	46,32–48,63	2,68	2,62–2,74
Biscoito salgado e salgadinho	42,00	40,54–43,47	2,41	2,33–2,49
Pães	33,05	31,83–34,27	1,94	1,86–2,01
Biscoitos doces	31,69	30,27–33,12	1,66	1,59–1,74
Frios e embutidos	29,99	29,15–30,83	1,71	1,66–1,75
Chocolate, sorvete e sobremesas industrializadas	29,16	27,66–30,66	1,48	1,41–1,55
Refrigerantes	25,80	24,81–26,78	1,40	1,34–1,45
Cachorro quente, hambúrgueres e sanduíches	22,03	20,56–23,5	1,18	1,1–1,26
Pizza	20,54	17,2–23,89	1,07	0,83–1,3
Bebidas lácteas	18,06	17,08–19,05	0,99	0,94–1,05
Pratos prontos ou semiprontos^e^	11,63	10,63–12,63	0,65	0,59–0,7
Salgados fritos ou assados	11,55	10,44–12,66	0,62	0,56–0,68
Sucos e outras bebidas artificiais	10,45	9,8–11,1	0,57	0,53–0,6
Molhos prontos	8,05	7,6–8,5	0,44	0,42–0,47
Bolos e tortas doces	7,37	6,7–8,03	0,40	0,36–0,43
Outros^f^	10,24	9,33–11,14	0,50	0,45–0,54
**Total**	**1.754,61**	**1.743,5–1.765,8**	**100,00**	

^a^Farinha de milho, aveia e outras.

^b^Inclui cogumelos, frutos do mar e outras carnes.

^c^Inclui leite de coco, féculas, vinagre, sal.

^d^Inclui conserva de legumes, conserva de leguminosas, conserva de peixe, castanhas, nozes e sementes açucaradas/salgadas e extrato de tomate.

^e^Inclui pratos de massa congelados/prontos para aquecer, macarrão, sopas e outros alimentos “instantâneos”.

^f^Inclui cereal matinal, queijos, bebidas alcoólicas destiladas e suplementos proteicos/calóricos.

Dentre os alimentos *in natura* ou minimamente processados, destaca-se o arroz, que correspondeu a 10,54% da energia total, seguido por carne bovina, com 7,46%; feijão, com 6,22%; e carne de aves, com 5,71%. A seguir, em ordem decrescente de contribuição para a energia total consumida, aparecem frutas (2,90%), leite (2,69%), macarrão (2,67%), carne suína (2,34%), raízes e tubérculos (2,08%) e verduras e legumes (1,72%) ( Tabela 1 ).

Dentre os ingredientes culinários, o grupo de maior contribuição para o aporte total de energia foi o de óleos vegetais (7,66%), seguido de açúcares (5,91%). Dentre os alimentos processados, o grupo de maior contribuição para o total de energia foi o de pães (7,86%), seguido pelos queijos (1,60%) ( Tabela 1 ).

Dentre os alimentos ultraprocessados, destaca-se a margarina, que representou 2,68% da energia total, seguida de biscoitos salgados e salgadinhos, com 2,41%; pães, com 1,94%; biscoitos doces, com 1,66%; e frios e embutidos, com 1,71%. A seguir, em ordem decrescente de contribuição para a energia total consumida, aparecem chocolate, sorvete e sobremesas industrializadas (1,48%), refrigerantes (1,40%) e cachorro-quente, hambúrgueres e sanduíches (1,18%) ( Tabela 1 ).

A Figura apresenta a participação dos alimentos ultraprocessados no total de energia consumida de acordo com as UF. De forma geral, as UF das regiões Sul e Sudeste apresentaram as maiores médias de participação de alimentos ultraprocessados no total de energia consumida, enquanto os estados do Norte e Nordeste apresentaram as menores. As maiores participações foram encontradas no Rio Grande do Sul (24,06% do total de energia consumida), seguido por Santa Catarina (23,23%) e São Paulo (22,38%). As menores participações foram no Piauí (12,58%), Maranhão (13,87%) e Tocantins (13,92%).

**Figura:** Porcentagem da participação de alimentos ultraprocessados no total de calorias consumidas pela população brasileira com 10 anos ou mais de idade, segundo Unidades da Federação. Pesquisa de Orçamentos Familiares, 2017–2018. Unidades Federativas: AM: Amazonas; AP: Amapá; BA: Bahia; CE: Ceará; DF: Distrito Federal; ES: Espírito Santo; GO: Goiás; MA: Maranhão; MG: Minas Gerais; MS: Mato Grosso do Sul; MT: Mato Grosso; PA: Pará; PB: Paraíba; PE: Pernambuco; PI: Piauí; PR: Paraná; RJ: Rio de Janeiro; RN: Rio Grande do Norte; RO: Rondônia; RR: Roraima; RS: Rio Grande do Sul; SC: Santa Catarina; SE: Sergipe; SP: São Paulo; TO: Tocantins.

A Tabela 2 apresenta a análise de associação entre as variáveis socioeconômicas e participação de alimentos ultraprocessados no total de energia consumida pela população brasileira com 10 ou mais anos de idade. Todas as variáveis incluídas na análise foram significativamente associadas com a participação de alimentos ultraprocessados no total de energia consumida. Tanto na análise bruta quanto na ajustada, a participação de alimentos ultraprocessados no total de energia foi significativamente maior no sexo feminino (em relação ao masculino), menor nas pessoas negras (em relação às brancas) e na área rural (em relação à urbana), além de diminuir com o aumento da idade e aumentar com a escolaridade e renda. Na análise bruta, a participação de alimentos ultraprocessados foi maior nas regiões Sudeste, Sul e Centro-Oeste em relação à região Norte, mas, na análise ajustada, a significância estatística não se manteve para a região Centro-Oeste.

**Tabela 2:** Participação de alimentos ultraprocessados no total de calorias consumidas pela população brasileira com 10 anos ou mais de idade, segundo variáveis socioeconômicas e demográficas. Pesquisa de Orçamentos Familiares, 2017–2018.

Variáveis	% de participação calórica de alimentos ultraprocessados					
	Média bruta	Coeficiente bruto	IC95%	Média ajustada	Coeficiente ajustado^a^	IC95%
**Sexo**						
Masculino	19,13	Ref.	-	19,10	Ref.	-
Feminino	20,21	1,08	0,73 a 1,43^c^	20,24	1,15	0,82 a 1,48^c^
**Idade** ^b^						
Adolescente	26,49	Ref.	-	29,07	Ref.	-
Adulto	19,46	-7,03	-7,61 a -6,44	18,85	-10,22	-10,82 a -9,61
Idoso	15,11	-11,38	-12,1 a -10,65^d^	15,41	-13,66	-14,42 a -12,91^d^
**Raça/cor**						
Branco	21,26	Ref.	-	20,15	Ref.	-
Negro (Pretos e Pardos)	18,44	-2,82	-3,36 a -2,28^c^	19,31	-0,84	-1,38 a -0,30^c^
Amarelo	22,46	1,20	-1,54 a 3,94	20,73	0,58	-1,64 a 2,79
Indígena	20,75	-0,51	-3,77 a 2,75	21,51	1,35	-1,46 a 4,16
**Anos de estudo**						
Menos de 4 anos	13,40	Ref.	-	16,43	Ref.	-
De 4 a 8 anos incompletos	18,25	4,84	4,3 a 5,3	17,68	1,26	0,76 a 1,75
De 8 a 12 anos incompletos	20,24	6,83	6,2 a 7,4	19,14	2,72	2,12 a 3,31
12 anos ou mais	22,17	8,77	8,1 a 9,4^d^	22,18	5,75	5,13 a 6,38^d^
**Quintos de renda familiar per capita**						
1	16,85	Ref.	-	17,30	Ref.	-
2	18,17	1,32	0,66 a 1,98	18,45	1,14	0,49 a 1,79
3	19,63	2,78	1,73 a 3,84	19,99	2,68	1,71 a 3,66
4	20,71	3,86	3,06 a 4,67	20,64	3,33	2,52 a 4,15
5	23,10	6,25	5,46 a 7,04^d^	22,10	4,80	3,92 a 5,67^d^
**Situação do domicílio**						
Urbano	20,55	Ref.	-	20,16	Ref.	-
Rural	14,65	-5,90	-6,43 a -5,36^c^	16,96	-3,20	-3,66 a -2,74^c^
**Região do país**						
Norte	17,52	Ref.	-	18,42	Ref.	-
Nordeste	17,35	-0,17	-0,9 a 0,56	18,84	0,42	-0,31 a 1,15
Sudeste	20,86	3,34	2,44 a 4,23^c^	20,11	1,69	0,76 a 2,61^c^
Sul	22,42	4,89	4,01 a 5,78^c^	21,76	3,34	2,41 a 4,27^c^
Centro-Oeste	18,65	1,13	0,24 a 2,02^c^	17,86	-0,56	-1,46 a 0,35

^a^Análise de regressão linear com todas as variáveis incluídas simultaneamente no modelo.

^b^Adolescente (10 a 19 anos), adulto (20 a 59 anos) e idoso (acima de 60 anos).

^c^p ≤ 0,005 na comparação com a categoria de referência.

^d^p de tendência linear < 0,001.

A contribuição de alimentos ultraprocessados para o total de energia aumentou significativamente nos dez anos entre as duas pesquisas (de 18,67% para 19,69%) ( Tabela 3 ).

**Tabela 3:** Evolução temporal da participação de alimentos ultraprocessados no total de energia consumida (%) pela população brasileira com 10 anos ou mais de idade segundo estratos socioeconômicos e demográficos. Pesquisas de Orçamentos Familiares 2008–2009 e 2017–2018.

	Participação no total de calorias consumidas (%)		Coeficiente	IC95%
	Média			
	2008–2009	2017–2018		
**Brasil**	18,67	19,69	1,02	0,69 a 1,35^b^
**Sexo**				
Masculino	17,54	19,13	1,59	1,1 a 2,08^b^
Feminino	19,73	20,21	0,49	0,04 a 0,94^b^
**Idade** ª				
Adolescente	25,10	26,49	1,39	0,55 a 2,23^b^
Adulto	18,12	19,46	1,34	0,94 a 1,74^b^
Idoso	13,14	15,11	1,97	1,25 a 2,69^b^
**Raça/cor**				
Branco	21,07	21,26	0,19	-0,32 a 0,69^b^
Negro (Pretos e Pardos)	16,39	18,44	2,04	1,61 a 2,48^b^
Amarelo	17,89	22,46	4,57	-0,23 a 9,37
Indígena	14,79	20,75	5,96	1,54 a 10,39^b^
**Anos de estudo**				
Menos de 4 anos	12,23	13,40	1,18	0,63 a 1,73^b^
De 4 a 8 anos incompletos	17,64	18,25	0,61	0,03 a 1,18^b^
De 8 a 12 anos incompletos	20,91	20,24	-0,67	-1,3 a -0,04^b^
12 anos ou mais	25,47	22,17	-3,30	-4,18 a -2,42^b^
**Quintos de renda familiar per capita**				
1	13,31	16,85	3,54	3,01 a 4,07^b^
2	16,56	18,17	1,61	0,93 a 2,28^b^
3	18,08	19,63	1,56	0,77 a 2,35^b^
4	20,66	20,71	0,06	-0,71 a 0,83
5	24,75	23,10	-1,65	-2,47 a -0,83^b^
**Situação do domicílio**				
Urbano	19,94	20,55	0,60	0,23 a 0,98^b^
Rural	12,22	14,65	2,43	2 a 2,87^b^
**Regiões**				
Norte	14,57	17,52	2,95	2,31 a 3,59^b^
Nordeste	14,24	17,35	3,11	2,71 a 3,51^b^
Sudeste	21,03	20,86	-0,17	-0,81 a 0,47
Sul	22,64	22,42	-0,22	-0,94 a 0,5
Centro-Oeste	17,77	18,65	0,87	0,01 a 1,75^b^

^a^Adolescente (10 a 19 anos), adulto (20 a 59 anos) e idoso (acima de 60 anos).

^b^p de heterogeneidade < 0,05.

Observou-se uma interação estatisticamente significativa entre o ano de estudo e as variáveis sexo, raça/cor, escolaridade, renda, situação do domicílio e região. O aumento da participação dos alimentos ultraprocessados no total de energia consumida foi estatisticamente significativo em ambos os sexos, mas mais expressivo em pessoas do sexo masculino (de 17,54% para 19,13%) do que do feminino (de 19,73% para 20,21%). Diferentemente, a participação dos alimentos ultraprocessados no total de energia consumida aumentou significativamente entre pessoas de raça/cor negra (de 16,39% para 18,44%) e indígena (de 14,79% para 20,75%), mas não entre pessoas brancas e amarelas ( Tabela 3 ).

O consumo de alimentos ultraprocessados aumentou significativamente entre as pessoas com até quatro e entre quatro e oito anos de estudo (de 12,23% para 13,40% e de 17,64% para 18,25%, respectivamente). Por outro lado, houve uma ligeira redução do seu consumo entre aqueles com oito a doze anos de estudo (de 20,91% para 20,24%) e uma redução de maior magnitude na faixa de escolaridade mais elevada (de 25,47% para 22,17%). De forma similar, o consumo de alimentos ultraprocessados aumentou significativamente nos três quintos mais baixos de renda familiar (de 13,31% para 16,85% no 1º, de 16,56% para 18,17% no 2º, e de 18,08% para 19,63% no 3º) e reduziu significativamente no quinto mais alto (de 24,75% para 23,10%), não se alterando no 4º quinto ( Tabela 3 ).

O aumento da participação dos alimentos ultraprocessados no total de energia consumida foi estatisticamente significativo na área rural e urbana, mas mais intensa na primeira (de 12,22% para 14,65%) do que na segunda (de 19,94% para 20,55%). Por fim, o consumo de alimentos ultraprocessados aumentou expressivamente nas regiões Norte (de 14,57% para 17,52%) e Nordeste (de 14,24% para 17,35%), elevou-se ligeiramente na região Centro-Oeste (de 17,77% para 18,65%), mas não se alterou no Sul e no Sudeste ( Tabela 3 ).

## DISCUSSÃO

Este estudo, utilizando dados recentes e representativos da população brasileira, mostrou que os alimentos ultraprocessados representaram cerca de 20% do total de energia consumida em 2017–2018. Este consumo, no entanto, variou de forma significativa segundo estratos socioeconômicos e demográficos e foi maior no sexo feminino, entre adolescentes, pessoas brancas, com maior renda e escolaridade e moradores de áreas urbanas e das regiões Sul e Sudeste. Além disso, os resultados demonstraram que houve um aumento médio de 5,5% no consumo de alimentos ultraprocessados no período de dez anos, sendo mais expressivo em pessoas negras e indígenas, moradores da área rural e das regiões Norte e Nordeste e nos grupos populacionais com menores níveis de escolaridade e renda.

A associação inversa entre o consumo de alimentos ultraprocessados e a idade, observada no Brasil, reflete um padrão global. Países como Reino Unido, Estados Unidos, Canadá, Chile, Colômbia e México^12^também apresentam maior consumo de alimentos ultraprocessados entre crianças e adolescentes, explicitando a vulnerabilidade desse grupo populacional à crescente exposição e à facilidade de acesso a esses alimentos. Por outro lado, os estudos não são tão consistentes no tocante às análises por condição socioeconômica. Estudos de base populacional realizados em países latino-americanos apresentaram resultados semelhantes aos observados no Brasil. No Chile, indivíduos residentes em áreas urbanas, na região metropolitana e com maior renda apresentaram consumo significativamente maior de alimentos ultraprocessados^13^. No México, o consumo de alimentos ultraprocessados aumentou com o nível econômico e educacional^15^. Na Colômbia, os indivíduos de áreas urbanas e com nível socioeconômico alto apresentaram ingestão calórica de 1,5 a 1,7 vezes maior de alimentos ultraprocessados em comparação com os de nível socioeconômico mais baixo e os residentes em regiões rurais^14^. Diferentemente, em países de mais alta renda, como Estados Unidos, Reino Unido e Canadá, são observadas apenas pequenas diferenças no consumo de alimentos ultraprocessados entre estratos populacionais. Isso reflete como esses alimentos, que representam mais de 50% do total de energia consumida por essas populações, permearam e atingiram todas as camadas sociais, padronizando os hábitos alimentares^12 , 16 , 17^.

Apesar da pequena diferença de magnitude, também observamos menor consumo de alimentos ultraprocessados entre pretos e pardos (em relação aos brancos), mesmo após ajuste para outras variáveis sociodemográficas. O consumo alimentar é resultado da interação de distintos fatores, sendo também influenciado por características culturais, históricas e psicológicas, que estão muito atreladas a questões étnico-raciais e ao racismo. Apesar de os nossos resultados indicarem que pretos e pardos apresentaram maior adesão à regra de ouro do Guia Alimentar para a População Brasileira, é importante pontuar que isso não representa necessariamente maior qualidade global da alimentação. Outros estudos já indicaram que essa diferença se deve, quase que absolutamente, ao maior consumo de alimentos básicos como arroz e feijão por pretos e pardos, mas o mesmo não acontece para outros alimentos *in natura* ou minimamente processados como frutas, legumes, verduras, nozes e sementes^18^.

As análises relativas à evolução temporal mostram que os segmentos socioeconômicos e demográficos que apresentaram menor consumo de alimentos ultraprocessados em 2017–2018 são justamente aqueles que apresentaram um aumento mais expressivo no período avaliado. Logo, o crescimento do consumo de alimentos ultraprocessados no país pode ser explicado pela expansão do seu acesso por grupos socialmente mais vulneráveis, o que se deve à redução dos preços relativos desses alimentos, à ampliação da sua oferta nos mais diversos locais de compras e à crescente penetração das indústrias transnacionais em áreas mais remotas do país^19^. Análises de dados do Sistema Nacional de Índices de Preços ao Consumidor mostram que, embora os alimentos ultraprocessados ainda sejam mais caros do que alimentos *in natura* ou minimamente processados e ingredientes culinários, seu preço vem se reduzindo, desde o início dos anos 2000, de forma paulatina e substancial^21^. Similarmente, nos últimos anos, observou-se um aumento da aquisição de alimentos em redes de supermercados e que estes oferecem uma maior concentração de alimentos ultraprocessados em comparação a outros lugares de compras mais tradicionais^22^. A propaganda direta e específica de alimentos ultraprocessados para comunidades de renda mais baixa também tem ajudado a acelerar seu crescimento nesses segmentos da sociedade^19 , 20^.

Por outro lado, observou-se uma tendência de estagnação do consumo de alimentos ultraprocessados em estratos socioeconômicos mais privilegiados. Uma hipótese para esse fenômeno é de que, no período mais recente, tenha ocorrido a ampliação da divulgação de informações sobre os malefícios dos alimentos ultraprocessados (principalmente de alguns tipos, como as bebidas açucaradas), informações essas mais acessíveis a pessoas de melhores condições socioeconômicas^23^. Vale lembrar também que o Guia Alimentar para a População Brasileira, que assertivamente recomenda que o consumo de alimentos ultraprocessados seja evitado, foi publicado em 2014 e repercutiu rapidamente em diversos veículos midiáticos.

O consumo de alimentos ultraprocessados vem crescendo em muitos países. Dados de aquisição domiciliar de alimentos também mostraram, por exemplo, que, no Canadá, a contribuição dos alimentos ultraprocessados aumentou de 24,4% em 1938–1939 para 54,9% em 2001 e, no México, de 10,5% em 1984 para 23,1% em 2016^24 , 25^. Mais recentemente, análises de bases de dados de vendas de alimentos no varejo em 80 países mostraram uma ascensão significativa nas vendas dos alimentos ultraprocessados entre 2002 e 2016, com particular aceleração entre os países de renda média, e que esse aumento foi positivamente associado à evolução temporal do índice de massa corporal das populações^26^.

A busca por estratégias para reduzir ou desacelerar a expansão do consumo de alimentos ultraprocessados é, portanto, mandatória. Dentre as medidas possíveis, incluem-se taxações e intervenções no preço de ultraprocessados, regulação de propagandas e promoções comerciais, em especial aquelas voltadas ao público infantojuvenil, e a adequação da rotulagem nutricional. Tais medidas devem ser aliadas a ações de educação alimentar e nutricional e políticas de estímulo à produção e comercialização de alimentos *in natura* e minimamente processados, visando sua maior acessibilidade por todos os segmentos populacionais^20^. A aparente queda do consumo de alimentos ultraprocessados no estrato populacional de mais alta renda (dado esse não registrado em nenhuma edição anterior da POF) pode nos dar algumas pistas de que a norma social em relação ao consumo desses alimentos venha se modificando, embora dados futuros sejam importantes para confirmar essa tendência.

Dentre os pontos fortes deste estudo, destacam-se: caráter rigorosamente probabilístico das amostras estudadas e representatividade nacional, assegurada pelo estudo de mais de 30 mil pessoas residentes nas áreas urbanas e rurais das várias regiões do país e coleta de dados de dois dias de consumo alimentar por meio de software validado, além da disposição de base de dados com mais de 1.200 itens alimentares. Por outro lado, este estudo apresenta limitações oriundas de vieses potenciais inerentes ao uso de inquéritos alimentares: subestimação/superestimação do consumo de certos grupos de alimentos, diferenças entre as receitas culinárias reais e as receitas padronizadas e diferenças entre a composição nutricional real dos alimentos consumidos e a composição indicada pela tabela de composição nutricional utilizada. Para minimizar parte desses vieses, os instrumentos de coleta foram pré-testados e validados, procedimentos de controle de qualidade foram realizados durante a coleta de dados e registros inconsistentes foram excluídos e substituídos com valores imputados. Além disso, a tabela de composição nutricional de alimentos utilizada foi construída especificamente para a população brasileira com rigoroso controle de qualidade. Por fim, outra possível limitação está relacionada aos diferentes métodos aplicados para coletar as informações de consumo alimentar nas duas pesquisas. Apesar disso, foi demonstrado que tal mudança pouco afetou a estimativa da composição da dieta e que é possível comparar os dois bancos com as estratégias de harmonização utilizadas^27^.

Em conclusão, este estudo descreveu a distribuição sociodemográfica e o crescimento do consumo de alimentos ultraprocessados no Brasil. Os segmentos populacionais que apresentaram menor consumo relativo desses alimentos em 2017–2018 são justamente os que demonstram um aumento mais expressivo na análise temporal, apontando para uma tendência de padronização nacional em um patamar de consumo mais elevado e, portanto, com aumento de riscos à saúde da população.
